# Sociodemographic determinants of health insurance enrolment and dropout in urban district of Ghana: a cross-sectional study

**DOI:** 10.1186/s13561-019-0241-y

**Published:** 2019-07-06

**Authors:** Eric Nsiah-Boateng, Justice Nonvignon, Genevieve Cecelia Aryeetey, Paola Salari, Fabrizio Tediosi, Patricia Akweongo, Moses Aikins

**Affiliations:** 1 School of Public Health, College of Health Sciences, University of Ghana, Accra, Ghana; 2 Research, Policy, Monitoring and Evaluation Directorate, National Health Insurance Authority, Accra, Ghana; 3 Swiss Tropical and Public Health Institute, Basel, Switzerland; 4 University of Basel, Basel, Switzerland

**Keywords:** Enrolment, Dropout, National Health Insurance, Urban district, Ghana

## Abstract

**Background:**

Earlier studies have found significant associations between sociodemographic factors and enrolment in the National Health Insurance Scheme (NHIS) in Ghana. These studies were mainly household surveys in relatively rural areas with high incidence of poverty. To expand the scope of existing evidence, this paper examines policy design factors associated with enrolment and dropout of the scheme in an urban poor district using routine secondary data.

**Methods:**

This study is a cross-sectional quantitative analysis of 2014–2016 NHIS enrolment data of the Ashiedu Keteke district office. Descriptive and multivariate logistic regression analyses were performed to examine sociodemographic factors associated with NHIS enrolment and dropout.

**Results:**

A total of 215,724 individuals enrolled in the NHIS over the period under study, of which 98,232 (46%) were new members. About 41% of existing members in 2014 dropped out of the NHIS in 2015 and 53% of those in 2015 dropped out in 2016. The indigents (core poor) are significantly more likely to enrol and to drop out of the NHIS. However, the males, informal sector employees, social security and national insurance trust (SSNIT) contributors, and the aged (70+ years) are significantly less likely to enrol in the NHIS but more likely to retain coverage.

**Conclusions:**

A considerable number of members are dropping out of the NHIS. The indigents in particular, are increasingly enrolling in and dropping out of the NHIS whilst the males, informal sector employees, SSNIT contributors and the aged are not enrolling as expected but increasingly retaining coverage. Policy reforms to ensuring continued growth towards realization of universal health coverage should take these factors into consideration.

**Electronic supplementary material:**

The online version of this article (10.1186/s13561-019-0241-y) contains supplementary material, which is available to authorized users.

## Background

Increasingly, social health insurance (SHI) is becoming a preferred health financing mechanism for protecting the poor against catastrophic healthcare expenditure in low- and middle-income countries (LMICs) [1, 2]. SHI is also seen as a promising means for addressing equity in healthcare and achieving overarching goal of universal health coverage (UHC) through pooling of resources and risks [3–5]. Evidence abounds that SHI enhances resource mobilization and access to healthcare for the poor and vulnerable populations [5–8]. However, Wipf and Garand [9] posits that without sufficiently high participation, voluntary health insurance schemes are likely to suffer from adverse selection and higher claims and administrative expenses, which may threaten their sustainability in the long run.

In 2003, Ghana introduced National Health Insurance Scheme (NHIS) to reduce out-of-pocket payments at the point of healthcare use [10–12]. The scheme is operational in 166 districts across the country, and has a population coverage of 10.8 million (36%) and over 4000 network of healthcare providers as of December 2018 [13]. It is financed by 2.5% levy on selected goods and services, two and a half percentage points of workers’ contributions to the Social Security and National Insurance Trust (SSNIT), contributions from employees in the informal sector of the economy, approved funds by parliament, resources accrued to the National Health Insurance Fund (NHIF) from investments, and support from donor partners [12].

As a pro-poor policy, groups considered vulnerable and too poor are exempted from paying premium to the scheme. These groups include persons below the age of 18 years, the elderly aged 70 years or older, SSNIT pensioners, pregnant women, indigent (or core poor), and beneficiaries of the Livelihood Empowerment Against Poverty (LEAP) programme [12, 14]. The LEAP programme was introduced in the year 2008 to reduce poverty through conditional cash transfer, and has covered over 74,000 households in 99 districts across the country as of November, 2013 [15, 16]. The National Health Insurance Authority (NHIA) which is the regulator of health insurance schemes in the country and an implementer of the NHIS, enrols LEAP beneficiaries in the scheme separately from the indigents. This is done in collaboration with the Ministry of Gender, Children and Social Protection (MoGCSP). The NHIA determines an expansion of coverage, partly through the inclusion of the poor and vulnerable population groups that are exempted [17–19]. Yet, despite the relatively low contributions and large groups of the population exempted from paying premium (close to 70% of the members), more than half of the population remains uninsured.

Literature show that enrolment and retention in SHI schemes are significantly associated with demand- and supply-side factors [20–23]. At the individual level, demand-side factors that are positively associated with enrolment include income, education, and age [19, 20, 24, 25]. Others are sex, marital status, chronic episode, and trust in the scheme management [20, 22, 25–27]. The supply-side factors are knowledge and understanding of the health insurance scheme, perception of quality of healthcare, and trust in the management of the scheme [20]. Factors that are positively associated with retention of coverage include education, household size, and trust in the scheme management [20, 28]. Other motivating factors for retention of coverage are knowledge and understanding of the scheme, healthcare quality, and receipt of benefits in the previous year [20]. However, inappropriate benefits package, cultural beliefs, affordability of premiums, distance to healthcare facility, area of residence and legal and policy frameworks to support SHI schemes are some of the barriers to enrolment [8, 14, 20, 26, 28–30]. Interaction of some of these demand and supply-side factors also influence enrolment and retention of coverage in social health insurance [14, 20, 26].

A number of studies have investigated factors influencing enrolment and dropout in SHI in LMICs using surveys and systematic reviews [14, 19–21, 28, 29]. This study adds to the existing evidence by examining sociodemographic determinants of health insurance enrolment in an urban poor district of Ghana. The study uses quantitative method to analyse routine secondary enrolment data of Ghana’s NHIS. It provides evidence to guide development of interventions to increase enrolment and sustain growth towards realization of UHC in the NHIS and other SHI schemes in LMICs.

## Methods

### Study design and setting

The design is a cross-sectional study of NHIS enrolment data for the period, 2014–2016. The selection of this period for the study was driven by reliability of the data. Membership data for the last quarter of 2016; however, was not available at the time of this study. The Ashiedu Keteke district in Ghana was used for the study because of its cosmopolitan nature. The district is the central business district of the Accra Metropolis and the smallest among the six sub-metropolitan districts (Additional file 1). However, it has one of the largest slums (Sodom and Gomorrah) in the city capital [31], which serves as dwelling place for migrants from the northern ecological zone of the country.

In the 2010 Population and Housing Census (PHC), the district had a population of 117,525 and an estimated daily commuter population of 200,000 [17, 32]. A district population of 62,360 (53%) were females; 32,410 (28%) were in the age bracket of 0–14 years; 80,362 (68%) were between 15 and 64 years; and 4753 (4%) were 65 years or older [32]. The district is also characterised by low socioeconomic status with majority of the inhabitants engaged in petty trading, fishing and fish-mongering. In 2013, about 104 individuals were beneficiaries of the LEAP programme in the district with a monthly cash transfer benefit of GHS24.00 (USD4.57) [33]. The district has one specialized hospital (Children’s hospital), a polyclinic, and a number of public and private clinics, and community pharmacies.

### Study population

Enrolment data of members of the Ashiedu Keteke district NHIA office was used for the study. The data covered a period of 3 years from 2014 to 2016 and were generated by the NHIA’s biometric system of registration. This system was introduced in the year 2014 to address challenges such as duplication, difficulty in generating reports, among others, associated with the previous non-biometric system. The biometric enrolment data obtained comprised characteristics including age, sex, and member category. Other characteristics were member number, year of entry, and enrolment type (new or renewal).

### Data collection and analysis

Data for the study were obtained from the district NHIA office using pre-designed template. Descriptive analysis was performed to determine the proportion of members by enrolment status, age, sex, and member category. Two multivariate logistic regression analyses were also conducted to determine associations between member characteristics and enrolment status. In the first multivariate analysis, the outcome variable (enrolment status) was assigned a discrete value of ‘1’ if an individual is enrolled as a new member and ‘0’ if an individual is enrolled as a renewed member. In the second analysis, the outcome variable was assigned a value of ‘1’ if an individual enrolled in 2014 as a new member but dropped out in 2015 and ‘0’ if an individual enrolled in 2014 as a new member and renewed membership in 2015. The explanatory variables were age, sex, and member category defined as being a person below the age of 18 years, indigent, informal sector employee (18–69 yrs), LEAP beneficiary, aged (70 + yrs), pregnant woman, SSNIT contributor and pensioner.

In both analyses, a person below the age of 18 years was used as a reference category for comparison to determine the odds of other member categories enrolling in the scheme. The reason is that persons below the age of 18 years are more susceptible to diseases, and are at the highest risk of poor health and death [34]. Thus, they are more likely to enrol and retain coverage in the NHIS by their parents. After testing for collinearity using Pearson’s R test, age was eliminated from the final model because it strongly correlated with member category, *r* = 0.62. The selection of member category over age for inclusion in the final model was informed by the nature of the enrolment data. Bootstrapping analytical technique was also applied to test the predictive power or consistency of the two regression models. This analytical method tests the accuracy of regression models by running its performance on subsets of the dataset over a number of times (replications) [35, 36]. Thus, it indicates how the predictive power or accuracy of a regression model is bounded. In this study, the performance of the two regression models were tested 1000 times through three subsets of the data: training, testing and validation [35, 36]. Stata version 13 and Microsoft excel 2016 was used to perform the analyses, and a conservative threshold of *p* < 0.05 was set to determine statistical significance.

## Results

### Nature of the enrolment data

Table 1 shows distribution of members by age, sex, and category over the same period. A total enrolment of 215,724 was recorded, of which 98,232 (46%) were new enrolments (not in table); 117,419 (54%) active members were in the age group of 18–69 years; 127,729 (59%) were females; and 81,549 (38%) belonged to the informal sector of the economy (not in table).

**Table 1:** Enrolment by age, sex, and member category from 2014 to 2016

Variable	2014		2015		2016 (Jan.–Sept.)	
	New (%)	Renewal (%)	New (%)	Renewal (%)	New (%)	Renewal (%)
Age						
< 18	17,145 (43.4)	13,194 (38.8)	18,273 (43.6)	17,746 (40.6)	6794 (40.5)	16,040 (40.3)
18–69	21,587 (54.6)	18,690 (55.0)	23,029 (54.9)	23,291 (53.4)	9705 (57.9)	21,117 (53.0)
70+	800 (2.0)	2124 (6.2)	633 (1.5)	2619 (6.0)	266 (1.6)	2670 (6.7)
Sex						
Female	24,022 (60.8)	19,729 (58.0)	24,545 (58.5)	25,383 (58.1)	9783(58.4)	24,267 (60.9)
Male	15,510 (39.2)	14,280 (42.0)	17,390 (41.5)	18,273 (41.9)	6982 (41.6)	15,560 (39.1)
Member category						
Child under 5 yrs	6307 (16.0)	2539 (7.5)	8365 (19.9)	5352 (12.3)	4207 (25.0)	5539 (13.9)
Child aged 5-17 yrs	9617 (24.3)	10,425 (30.7)	9105 (21.7)	12,353 (28.3)	2607 (15.6)	10,839 (27.2)
Indigent	1334 (3.4)	404 (1.2)	1468 (3.5)	451 (1.0)	33 (0.2)	92 (0.2)
Informal sector employee	14,815 (37.5)	12,653 (37.2)	15,972 (38.0)	16,572 (38.0)	6454 (38.5)	15,083 (37.9)
LEAP beneficiary	26 (0.1)	37 (0.1)	32 (0.1)	52 (0.1)	60 (0.4)	48 (0.1)
Aged (≥70 yrs)	756 (1.9)	2153 (6.3)	586 (1.4)	2534 (5.8)	240 (1.4)	2492 (6.3)
Pregnant woman	5256 (13.3)	1171 (3.4)	5031 (12.0)	1211 (2.8)	2761 (16.5)	1793 (4.5)
SSNIT contributor	1393 (3.5)	4479 (13.2)	1364 (3.3)	4975 (11.4)	395 (2.3)	3802 (9.5)
SSNIT pensioner	28 (0.1)	148 (0.4)	12 (0.1)	156 (0.4)	8 (0.1)	139 (0.3)
Total	39,532 (53.8)	34,009 (46.2)	41,935 (49.0)	43,656 (51.0)	16,765 (29.6)	39,827 (70.4)

*LEAP* Livelihood Empowerment Against Poverty, *SSNIT* Social Security and National Insurance Trust

**Fig. 1:**
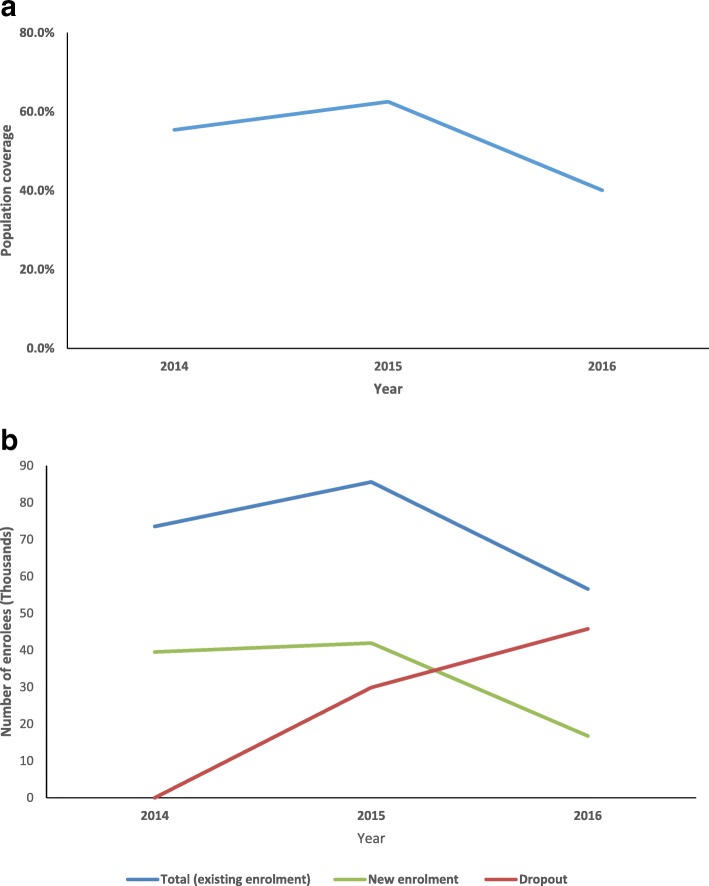
**a** Trend in population coverage, 2014–2016. **b** Trends in enrolment types, 2014–2016

Over the study period, population coverage of the scheme increased from 55% to 63% between 2014 and 2015 and declined to 40% in September 2016 (Fig. 1a). Trends in enrolment also show that total enrolment (existing enrolment) and new enrolment assumed a downward trajectory after the base year (2014) (Fig. 1b). Existing enrolment increased by 16% from 73,541 members in 2014 to 85,591 members in 2015, and then declined by 34% to 56,592 members in September 2016. Likewise, new enrolment increased by 6% from 39,532 members in 2014 to 41,935 members in 2015, and then declined by 60% to 16,764 members in September 2016. These downward trends resulted in an increase in dropouts by 41% (29,885) between 2014 and 2015 and 53% (45,764) between 2015 and September 2016.

### Relationship between member characteristics and NHIS enrolment

Several individual and policy-related characteristics were associated with enrolment in the NHIS. In the pooled data, results of the multivariate analysis showed that being an indigent (OR = 2.48, 95% CI: 2.25–2.73) was significantly associated with high enrolment in the NHIS compared to persons below the age of 18 years (Table 2). Similarly, by combining individual and policy-related characteristics, being a male SSNIT pensioner (OR = 4.00, 95% CI: 1.94–8.24), male informal sector employee (OR = 2.49, 95% CI: 2.39–2.58), male SSNIT contributor (OR = 2.02, 95% CI: 1.85–2.19), male aged 70 years or older (OR = 1.61, 95% CI: 1.43–1.80) or male indigent (OR = 1.43, 95% CI: 1.22–1.66) was significantly associated with high enrolment in the scheme relative to a male below the age of 18 years.

**Table 2:** Multivariate logistic regression model estimates for NHIS enrolment

Variable	2014	2015	2016	Pooled (2014–2016
	OR (95% CI)	OR (95% CI)	OR (95% CI)	OR (95% CI)
Member category				
Persons below 18 yrs	1.00			
Indigent	1.99 (1.72–2.32)***	2.17 (1.89–2.49)***	0.65 (0.38–1.09)	2.48 (2.25–2.73)***
Informal sector employee (18–69 yrs)	0.59 (0.56–0.61)***	0.56 (0.54–0.58)***	0.50 (0.48–0.53)***	0.57 (0.56–0.58)***
LEAP beneficiary	0.52 (0.29–0.92)**	0.49 (0.29–0.87)**	1.59 (1.02–2.49)**	0.72 (0.53–0.97)**
Aged (≥70 yrs)	0.21 (0.19–0.24)***	0.14 (0.13–0.16)***	0.14 (0.12–0.17)***	0.17 (0.16–0.19)***
SSNIT contributor	0.15 (0.14–0.17)***	0.16 (0.14–0.18)***	0.13 (0.11–0.16)***	0.16 (0.15–0.17)***
SSNIT pensioner	0.07 (0.03–0.15)***	0.01 (0.00–0.08)***	0.05 (0.01–0.19)***	0.04 (0.02–0.08)***
Sex				
Female	1.00			
Male	0.69 (0.66–0.72)***	0.68 (0.66–0.71)***	0.69 (0.65–0.72)***	0.69 (0.68–0.71)***
Member category and sex interaction				
Male Indigent	1.27 (1.00–1.59)**	1.73 (1.39–2.17)***	1.17 (0.52–2.61)	1.43 (1.22–1.66)***
Male Informal sector employee (18–69 yrs)	2.09 (1.96–2.23)***	2.53 (2.39–2.69)***	3.39 (3.14–3.67)***	2.49 (2.39–2.58)***
Male LEAP beneficiary	0.39 (0.09–1.59)	1.04 (0.42–2.62)	3.36 (1.49–8.93)**	1.38 (0.81–2.36)
Male Aged (≥70 yrs)	1.22 (1.02–1.46)**	2.09 (1.74–2.52)***	1.84 (1.39–2.43)***	1.61 (1.43–1.80)***
Male SSNIT contributor	1.88 (1.64–2.14)***	2.01 (1.76–2.29)***	2.22 (1.78–2.76)***	2.02 (1.85–2.19)***
Male SSNIT pensioner	2.82 (1.12–7.06)**	10.65 (1.34–84.58)**	4.05 (0.79–20.76)*	4.00 (1.94–8.24)***
_cons	1.76	1.40 (1.37–1.44)***	0.61 90.59_0.63)***	1.21 (1.19–1.23)***
Number of obs	73,541	85,591	56,592	215,724
LR chi2 (16)	4714.72	5483.40	3116.40	13,154.54
Prob > chi2	0.0000	0.0000	0.0000	0.0000
Log likelihood	−48,409.791	−56,568.159	−32,829.792	− 142,090.29
Pseudo R2	0.0464	0.0462	0.0453	0.0442

*OR* odds ratio; *p < 0.05; **p < 0.01; ***p < 0.001; *LEAP* Livelihood Empowerment Against Poverty, *SSNIT* Social Security and National Insurance Trust

However, being a male (OR = 0.69, 95% CI: 0.68–0.71), informal sector employee (OR = 0.57, 95% CI: 0.56–0.58), aged (OR = 0.17, 95% CI: 0.16–0.19), SSNIT contributor (OR = 0.16, 95% CI: 0.15–0.17), SSNIT pensioner (OR = 0.04, 95% CI: 0.02–0.08) or LEAP beneficiary (OR = 0.72, 95% CI: 0.53–0.97) was significantly associated with low enrolment in the NHIS. Similar trends were also observed in each study period. Results of the bootstrapping model to test the predictive power of the logistic regression model shows a narrower 95% CI (0.344–0.348) of the Area under ROC Curve (AUC), indicating high accuracy of the model in predicting enrolment in the NHIS (Additional file 2).

### Relationship between member characteristics and NHIS dropout

Results of the logistic regression shows that being an indigent (OR = 2.27, 95% CI: 1.68–3.07) was significantly associated with dropping out of the NHIS (Table 3). Likewise, an interaction between individual and policy-related characteristics shows that being a male informal sector employee (OR = 2.47, 95% CI: 2.17–2.79); male aged (OR = 2.10, 95% CI:1.47–2.64); or male SSNIT contributor (OR = 1.97, 95% CI: 1.47–2.64) was significantly associated with dropping out of the scheme. However, being a male (OR = 0.61, 95% CI: 0.56–0.66); informal sector employee (OR = 0.77, 95% CI: 0.71–0.83); SSNIT contributor (OR = 0.55, 95% CI: 0.44–0.69) or aged 70 years or older (OR = 0.33, 95% CI: 0.27–0.39) was significantly associated with retention of membership in the scheme. A test of predictive power or performance of the model using bootstrapping method (resampling) shows a narrower bias 95% CI (0.33–0.34), suggesting accurate estimates of the predictors (Additional file 3).

**Table 3:** Multivariate logistic regression model estimates for NHIS dropout

Variable	Odds Ratio	(95% C.I)
Member category		
Persons below 18 yrs	1.00	
Indigent	2.27	(1.68–3.07)***
Informal sector employee (18–69 yrs)	0.77	(0.71–0.83)***
Aged (≥70 yrs)	0.33	(0.27–0.39)***
SSNIT contributor	0.55	(0.44–0.69)***
Sex		
Female	1.00	
Male	0.61	(0.56–0.66)***
Member category and sex interaction	1.00	
Male indigent	1.19	(0.77–1.86)
Male informal sector employee (18–69 yrs)	2.47	(2.17–2.79)***
Male Aged (≥70 yrs)	2.09	(1.47–2.96)***
Male SSNIT contributor	1.97	(1.47–2.64)***
_cons	7.18	(6.81–7.56)***
Number of obs	39,532	
LR chi2 (14)	433.28	
Prob > chi2	0.0000	
Log likelihood	−16,048.971	
Pseudo R2	0.0133	

*OR* odds ratio; ***p < 0.001; *SSNIT* Social Security and National Insurance Trust

## Discussion

This study examined individual characteristics and policy design features that influence NHIS enrolment and dropout in an urban poor district of Ghana. The findings show that the number of people taking up new membership in the NHIS are declining and existing members are increasingly dropping out of the scheme; consequently, population coverage has assumed a downward trend. Sociodemographic factors such as being a male, indigent, informal sector employee, social security contributor or pensioner, or an aged (70+ years) is associated with NHIS enrolment and dropout. However, there are no substantial variations in the sociodemographic factors associated with enrolment in each year of the study period.

The increasing dropout of the NHIS and the resultant decline in population coverage is due to both demand and supply-side factors. Demand-side factors including the inability of the National Health Insurance Authority (NHIA) to ensure continuous availability of materials to produce membership cards in the period under study (2014–2016) might have contributed to the downward trend in coverage. Anecdotal reports show that this situation created inconvenience for people to enrol in the NHIS because they had to queue for long hours at the district offices or registration centres. Clearly, those who were dissatisfied and could not wait for long hours to enrol would leave the offices or registration centres. Besides, reported cases of unauthorised out-of-pocket payment at the healthcare provider sites are plausible causes of the decreasing population coverage. Evidence show that these factors negatively affect enrolment in social health insurance programmes [14, 20, 29, 31, 37]. Although the NHIA has recently introduced an innovative electronic membership renewal system for members whose cards have expired to renew their membership through the use of mobile money, efforts are needed to deepen the public knowledge of the NHIS especially the benefit package. There is also the need for policy makers to address reported cases of illegal charges at the healthcare facilities. Our finding is consistent with similar studies on the NHIS [31, 38, 39].

Findings of the study also show that the indigents are significantly more likely to enrol and to drop out of the NHIS relative to persons below the age 18 years. The increased enrolment among the indigents is related to the fact that most of them are identified and enrolled in the scheme by donor partners such as the World Bank and other philanthropic organisations. The increased collaborative effort between the NHIA and Ministry of Gender, Children and Social Protection (MoGCSP) in recent years, to increase the number of poor and vulnerable groups in the NHIS might have also accounted for the remarkable enrolment of indigents in the scheme. The high dropout of enrolment by the indigents; however, suggests that the same efforts are not being made by the two institutions to ensuring continued retention of coverage for this subgroup after enrolling them in the scheme. Although, indigents are exempted from paying premium, the mandatory payment of a membership card processing fee of GHS5.00 (USD0.95) during renewals is a significant barrier as found in previous studies [14, 28]. This annual renewal processing fee is almost half of the national daily minimum wage of GHS10.65 (USD2.03) [40] but constitutes less than a percent of the annual minimum wage.

Our findings; however, show that the informal sector employees are less likely to enrol in the scheme but more likely to retain coverage. This may be attributed to the fact that majority of the them believe that they have low healthcare needs or are mostly healthy [14, 31]. Similarly, the SSNIT contributors are significantly less likely to enrol in the scheme but more likely to retain coverage. The plausible reason is that these are formal sector employees who receive consistent income; therefore, they can afford to pay out-of-pocket for healthcare or take up private health insurance [31]. These findings are consistent with earlier studies in Ghana [14, 29] but contradict a study in Kenya which found that individuals participating in National Social Security Fund, saving schemes, and community-based saving groups were significantly more likely to have public health insurance [41]. Persons aged 70 years or older are also less likely to enrol in the scheme but more likely to retain coverage, which corroborates an earlier study [42]. The plausible explanation is that this subpopulation has more healthcare needs [22, 43]; therefore, would take up health insurance to avoid unexpected catastrophic healthcare expenditure.

Moreover, the study reveals that males are significantly less likely to enrol in the NHIS relative to females but more likely to retain coverage, which corroborates previous studies [20, 26, 41]. However, interaction between individual and policy-related characteristics shows that male informal sector employees, male SSNIT contributors, male SSNIT pensioners and males aged 70 years or older are significantly more likely to enrol in the NHIS. This occurrence might be due to a combination of factors including adverse selection, especially of the aged and SSNIT pensioners, who usually have more healthcare needs but inadequate incomes [22]. Thus, they are most likely to seek financial protection under the scheme against their healthcare cost as emphasised earlier.

One significant implication of our findings is that the high dropout rate of the NHIS, coupled with the large number of members exempted from paying premium to the scheme, has the potential to pose huge financial burden on the scheme, which could threaten its sustainability. For instance, this phenomenon could reduce risk pooling and financial risk protection for members of the scheme, particularly the poor and vulnerable, and eventually derail progress towards attainment of UHC. Policy makers need to enforce the mandatory enrolment provision in the law governing operations of the scheme. This can be done by making enrolment in the scheme a prerequisite for obtaining certain services such as driving licence and employment in both public and private institutions, as is the case for enrolment into secondary and tertiary educational institutions in the country after one obtains offer of admission.

### Limitations

Some limitations of the study are worth mentioning. First, the administrative data lack important demand-side information on income level, education, household size, marital status, health status and length of enrolment, found in literature as factors associated with enrolment in health insurance schemes. The data also lack important supply-side factors including population-to-doctor ratio, availability of healthcare facilities, and distance to healthcare facility. Although, this situation limited the explanatory variables for determining why certain groups are more likely to enrol in the scheme, the multivariate logistic regression models show consistencies in their predictive powers when bootstrapping (resampling) was applied to test their performances in predicting the outcome. The findings of the study also show consistency with a number of similar studies in LMICs, indicating reliability of the study findings for informed policy decision-making.

Secondly, the study could not employ panel data analysis to account for heterogeneity and other unobservable effects in the insured data over the 3-year period because the data were unbalanced. Available data for the year 2016 were less than 1 year (January–September). Although, a Breusch-Pagan Lagrange Multiplier (LM) test showed variances across the members (insured), these variances were too small (close to zero) for a panel analysis. Again, lack of data for the last quarter of 2016 at the time of this study made it impossible to completely track the odds of new members in 2014 and 2015 dropping out of the scheme in 2016. Lastly, the use of enrolment data from one district office of the NHIA may limit generalization of the results to the national member population of the scheme. The NHIS member population of the study area represents only a percent of the national active member population. Thus, transferability of the results to other urban districts needs to take into account the low socioeconomic status in this study area and other related factors.

## Conclusions

The study reveals that indigents are enrolling in the scheme more than renewing their membership whilst the males, informal sector employees, SSNIT contributors and the aged (70 years or older) are renewing their membership more than new members enrolling. Policy reforms seeking to achieve and sustain UHC should consider these factors especially using targeted advocacy and promotional health education to improve enrolment in the NHIS. Further research should investigate low enrolment in the NHIS among informal sector employees and the vulnerable groups so that appropriate intervention can be designed and implemented to sustain the scheme. The proposed research should take into account individual, NHIS, and healthcare provider related factors to give a comprehensive view of factors influencing enrolment in the NHIS.

## Additional files

Additional file 1: Map of Accra Metropolis showing Ashiedu Keteke district; adopted from https://www.hensongeodata.com/map/4/. (JPG 139 kb)

Additional file 2: Bootstrap (resampling) model estimates for NHIS enrolment. (DOCX 133 kb)

Additional file 3: Bootstrap (resampling) model estimates for NHIS dropout. (DOCX 133 kb)

## Data Availability

The datasets generated and/or analysed during the current study are not publicly available due to confidential information of the insured but are available from the corresponding author on reasonable request.

## Abbreviations

AUC: Area under ROC Curve
LEAP: Livelihood Empowerment against Poverty
LM: Breusch-Pagan Lagrange Multiplier
LMICs: Low-and Middle-Income Countries
MoGCSP: Ministry of Gender, Children and Social Protection
NHIA: National Health Insurance Authority
NHIF: National Health Insurance Fund
NHIS: National Health Insurance Scheme
PHC: Population and Housing Census
ROC: Receiver Operating Characteristic
SHI: Social Health Insurance
SSNIT: Social Security and National Insurance Trust
UHC: Universal Health Coverage
