# Supplementary figures and images for: Sociodemographic determinants of health insurance enrolment and dropout in urban district of Ghana: a cross-sectional study
Source: Health Econ Rev. 2019 Jul 6;9:23. doi: 10.1186/s13561-019-0241-y (PMC6734452; doi:10.1186/s13561-019-0241-y)

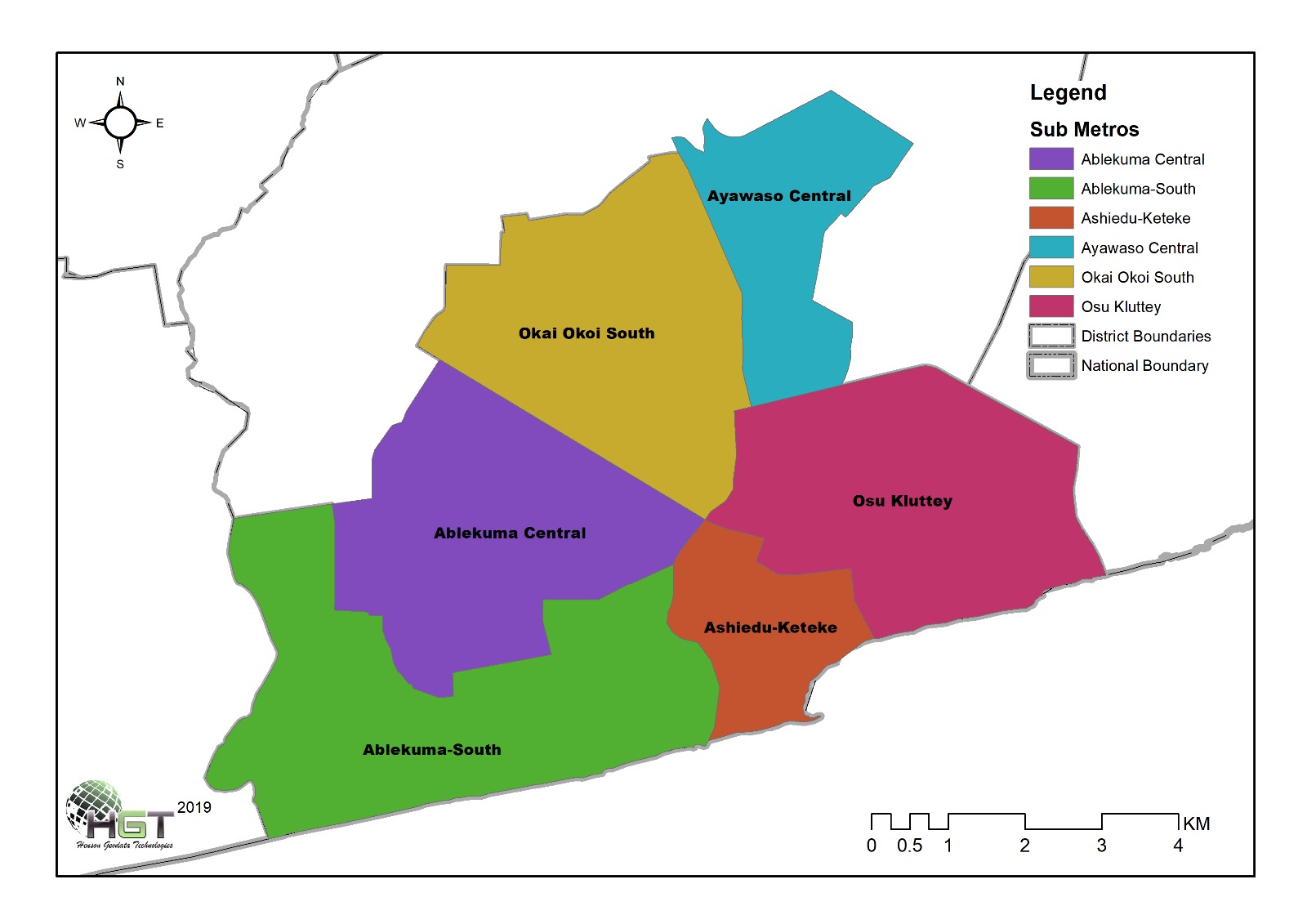

Supplement: Supplementary file 1 — Map of Accra Metropolis showing Ashiedu Keteke district; adopted from https://www.hensongeodata.com/map/4/. (JPG 139 kb) [file 13561_2019_241_MOESM1_ESM.jpg]
