# Supplementary material for: Sociodemographic determinants of health insurance enrolment and dropout in urban district of Ghana: a cross-sectional study
Source: Health Econ Rev. 2019 Jul 6;9:23. doi: 10.1186/s13561-019-0241-y (PMC6734452; doi:10.1186/s13561-019-0241-y)
Supplement: Supplementary file 2 — Bootstrap (resampling) model estimates for NHIS enrolment. (DOCX 133 kb) [file 13561_2019_241_MOESM2_ESM.docx]

**Additional file 2:** Bootstrap (resampling) model of enrolling in the NHIS

Area under ROC Curve (AUC); (N): Normal confidence interval; (P): Percentile confidence interval; (BC): Bias corrected confidence interval
